# *Pnma*-BN: Another Boron Nitride Polymorph with Interesting Physical Properties

**DOI:** 10.3390/nano7010003

**Published:** 2016-12-28

**Authors:** Zhenyang Ma, Zheng Han, Xuhong Liu, Xinhai Yu, Dayun Wang, Yi Tian

**Affiliations:** Tianjin Key Laboratory for Civil Aircraft Airworthiness and Maintenance, Civil Aviation University of China, Tianjin 300300, China; hanzhengcauc@163.com (Z.H.); hxliu@126.com (X.L.); xhyu11987@163.com (X.Y.); dayunwangcauc@163.com (D.W.); ty_cauc@163.com (Y.T.)

**Keywords:** BN polymorph, mechanical properties, anisotropic properties, electronic properties

## Abstract

Structural, mechanical, electronic properties, and stability of boron nitride (BN) in *Pnma* structure were studied using first-principles calculations by Cambridge Serial Total Energy Package (CASTEP) plane-wave code, and the calculations were performed with the local density approximation and generalized gradient approximation in the form of Perdew–Burke–Ernzerhof. This BN, called *Pnma*-BN, contains four boron atoms and four nitrogen atoms buckled through *sp*^3^-hybridized bonds in an orthorhombic symmetry unit cell with Space group of *Pnma*. *Pnma*-BN is energetically stable, mechanically stable, and dynamically stable at ambient pressure and high pressure. The calculated Pugh ratio and Poisson’s ratio revealed that *Pnma*-BN is brittle, and *Pnma*-BN is found to turn brittle to ductile (~94 GPa) in this pressure range. It shows a higher mechanical anisotropy in Poisson’s ratio, shear modulus, Young’s modulus, and the universal elastic anisotropy index *A*^U^. Band structure calculations indicate that *Pnma*-BN is an insulator with indirect band gap of 7.18 eV. The most extraordinary thing is that the band gap increases first and then decreases with the increase of pressure from 0 to 60 GPa, and from 60 to 100 GPa, the band gap increases first and then decreases again.

## 1. Introduction

In recent years, with the development of technology the interest in theoretical design and experimental synthesis of new superhard materials has increased. Such materials are in great demand in material science, electronics, optics, and even jewelry. Usually, borides, nitrides, and the covalent compounds of light elements (B, Be, O, C, N, etc.) are regarded as candidates of superhard materials [1,2,3,4,5]. Among these materials, boron nitrides are a typical group. c-BN is a superhard material. Boron nitride has various polymorphs, which are similar to structural modifications of carbon. Boron nitride (BN) can stably exist in many polymorphs because B and N atoms can bind together by *sp*^2^ and *sp*^3^ hybridizations. Hexagonal boron nitride (h-BN) is a graphite-like layered structure of the ABAB type, where each layer is rotated with respect to the previous one [6]. Also, there is a range of phases, usually referred to as turbostratic boron nitride (t-BN) [7,8], which are located between highly ordered h-BN and an amorphous material. Besides the well-known cubic diamond-like phase (c-BN) [9], wurtzite-like phase (w-BN) [7], layered graphite-like phase (h-BN or r-BN) [6,10,11], BN nanosheet [12], and BN nanotubes (BNNTs) [13], many new BN polymorphs have been experimentally prepared or theoretical predicted, including P-BN [14], BC_8_-BN [15], T-B*_x_*N*_x_* [16], Z-BN [17], I-BN [18], cT_8_-BN [19], B_4_N_4_ [20], o-BN [21], bct-BN [22], zeolite-like microporous BN [23,24], turbostratic BN [25], and BN fiber [10].

Dai et al. [23] found two types of highly stable porous BN materials using a Particle Swarm Optimization (PSO) algorithm as implemented in Crystal structure AnaLYsis by Particle Swarm Optimization (CALYPSO) code, and the first-principles calculations are utilized in properties calculations. In particular, type-II BN material lz3-BN with a relatively large pore size appears to be highly favorable for hydrogen adsorption as the computed average hydrogen adsorption energy is very close to the optimal adsorption energy suggested for reversible adsorptive hydrogen storage at room temperature. Li et al. [26] performed a systematic search for stable compounds in the BN system. They found a new stable N-rich compound with stoichiometry of B_3_N_5_ (*C*222_1_ phase), which at ambient pressure has a layered structure with freely rotating N_2_ molecules intercalated between the layers. Therefore, the *C*222_1_ phase is a potential high-energy-density material. Calculations also revealed *C*222_1_-B_3_N_5_ to be superhard.

In this paper, structural, mechanical, electronic properties, and stability of *Pnma*-BN were first studied using first-principles calculations by Cambridge Serial Total Energy Package (CASTEP) plane-wave code, and the calculations were performed with the local density approximation and generalized gradient approximation in the form of Perdew–Burke–Ernzerhof. *Pnma*-BN with space group 62 has four-, six-, and eight-membered rings, it is a three-dimensional structure, which is different from that of layered two-dimensional material (for example: h-BN).

## 2. Computational Methods

The total energy calculations were performed using density functional theory (DFT) with the Perdew–Burke–Ernzerhof (PBE) exchange correlation in the framework of the generalized gradient approximation (GGA) [27] and Ceperley and Alder data as parameterized by Perdew and Zunger (CA-PZ) in the framework of the local density approximation (LDA) [28] as implemented in the Cambridge Serial Total Energy Package (CASTEP) plane-wave code [29]. The equilibrium crystal structures were achieved by utilizing geometry optimization in the Broyden–Fletcher–Goldfarb–Shanno (BFGS) [30] minimization scheme. The interactions between the ionic core and valence electrons were described by the ultrasoft pseudo-potential [31], and the 2*s*^2^2*p*^1^ and 2*s*^2^2*p*^3^ were considered as valence electrons for B and N, respectively. The plane-wave basis set was truncated with an energy cutoff of 500 eV, and the Brillouin zone integration was generated using Monkhorst-Pack *k*-point meshes [32] with a high-quality grid of 0.025 Å^−1^ (8 × 15 × 9) for total-energy and elastic constants calculations, respectively. The elastic constants were calculated by the strain–stress method, which has been successfully utilized previously [33,34]. The bulk modulus, shear modulus, Young’s modulus, and Poisson’s ratio were estimated via Voigt–Reuss–Hill approximation [35,36,37].

## 3. Results and Discussion

### 3.1. Structural Properties

**Figure 1:**
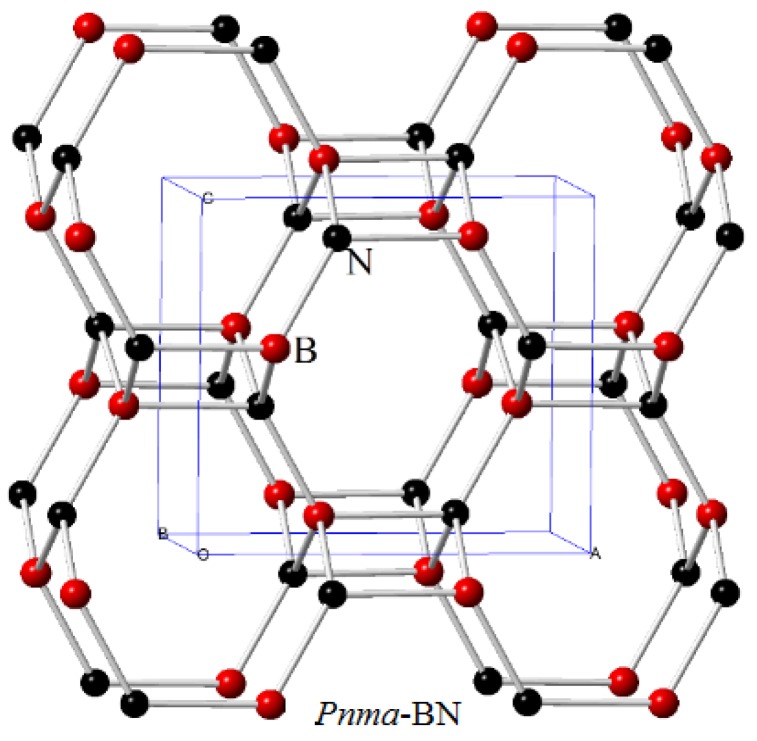
Unit cell crystal structures of BN in *Pnma* structure.

*Pnma*-BN adopts a *Pnma* symmetry with atoms occupying the B (−0.1601, 0.2500, 0.4087) and N (0.1773, 0.2500, 0.3909) positions. *Pnma*-BN has the lattice parameters *a* = 4.890 Å, *b* = 2.589 Å, *c* = 4.284 Å with GGA at ambient pressure. The crystal structure of *Pnma*-BN is shown in Figure 1. From Figure 1, *Pnma*-BN shares the configurations of four-, six-, and eight-membered *sp*^3^-bonded rings, and it is a three-dimensional structure. The calculated lattice parameters of *Pnma*-BN, *Pbca*-BN, and $F\bar{4}3m$-BN (c-BN) are listed in Table 1. For *Pnma*-BN, *Pbca*-BN, and $F\bar{4}3m$-BN, the calculated lattice parameters are in excellent agreement with the reported calculated results [38,39,40], and the calculated lattice parameters of $F\bar{4}3m$-BN are in excellent agreement with the experimental results [41]. With the pressure increasing to 50 GPa, the B atoms’ positions change to (−0.1618, 0.2500, 0.4004), and the N atoms positions change to (0.2046, 0.2500, 0.4126); while under 100 GPa, the B atoms’ positions change to (−0.1621, 0.2500, 0.3950), the N atoms’ positions change to (0.2219, 0.2500, 0.4256). Compared to the boron atoms, the change of atom positions of the nitrogen atoms are much larger than that of the boron atoms.

**Figure 2:**
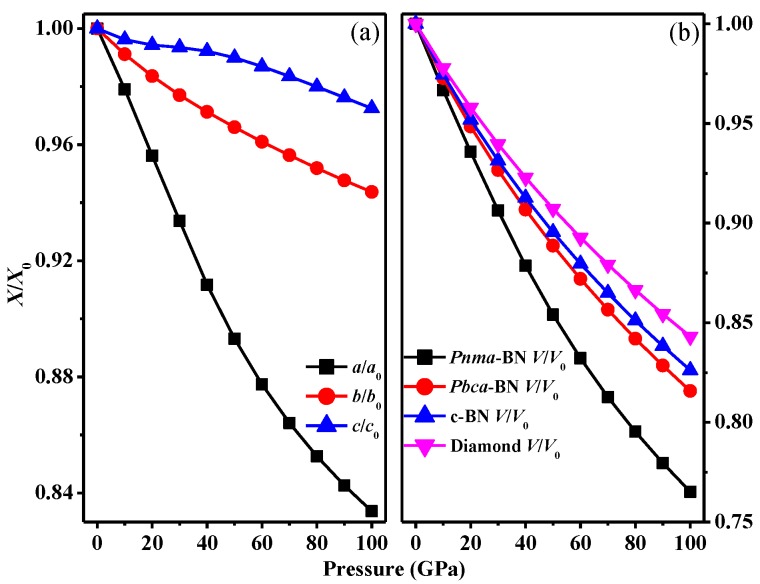
The lattice constants *a*/*a*_0_, *b*/*b*_0_, *c*/*c*_0_ compression as functions of pressure for *Pnma*-BN (**a**), and primitive cell volume *V*/*V*_0_ for *Pbca*-BN, *Pnma*-BN, c-BN, and diamond (**b**).

The structural properties, as well as the dependences of the normalized lattice parameters and volume on pressure up to 100 GPa for *Pnma*-BN, are shown in Figure 2. From Figure 2a, the lattice parameters of *Pnma*-BN decrease with increasing pressure, while for lattice parameter *c*, it decreases with a slightly smaller speed as pressure increases from 20 GPa to 40 GPa than other ranges. We noted that, when the pressure increases, the compression along the *c*-axis is much larger than those along the *a*-axis and *b*-axis in the basal plane. From Figure 2a, we can also easily see that the compression of *c*-axis is the most difficult. For the volumes on pressure up to 100 GPa of *Pnma*-BN, *Pbca*-BN, $F\bar{4}3m$-BN, and diamond, it can be easily seen that the compression of diamond is the most difficult. From Figure 2b, it can be seen that the incompressibility of *Pbca*-BN and $F\bar{4}3m$-BN is better than *Pnma*-BN. So we can expect the bulk modulus of *Pnma*-BN is smaller than that of *Pbca*-BN and $F\bar{4}3m$-BN. For $F\bar{4}3m$-BN, the calculated lattice parameters using GGA level are closer than that of experimental results (see Table 1), so we use the results of elastic constants and elastic modulus of *Pnma*-BN within the GGA level in this paper.

### 3.2. Stability

The orthorhombic phase has nine independence elastic constants *C_ij_* (*C*_11_, *C*_12_, *C*_13_, *C*_22_, *C*_23_, *C*_33_, *C*_44_, *C*_55_, *C*_66_), and the elastic constants and elastic modulus of *Pnma*-BN are listed in Table 2. The criteria for mechanical stability of the orthorhombic phase are given by [42]:

(1) $$C_{ij}>0,i,j=1\sim 6$$

(2) $$[C_{11}+C_{22}+C_{33}+2(C_{12}+C_{13}+C_{23})]>0$$

(3) $$(C_{11}+C_{22}-2C_{12})>0$$

(4) $$(C_{11}+C_{33}-2C_{13})>0$$

(5) $$(C_{22}+C_{33}-2C_{23})>0$$

**Figure 3:**
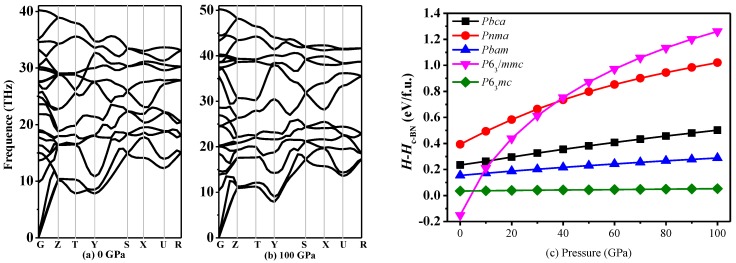
The phonon spectra of *Pnma*-BN at 0 GPa (**a**) and 100 GPa (**b**); Mixing enthalpy Δ*H* of BN alltropes calculated using PBE (**c**).

The calculated elastic constants under ambient pressure and high pressure of *Pnma*-BN indicated that it is mechanically stable because of the satisfaction of the mechanical stability criteria. To confirm the stability of *Pnma*-BN, their dynamical stabilities should also be studied under ambient pressure and high pressures. Thus, the calculated the phonon spectra for *Pnma*-BN at 0 and 100 GPa are shown in Figure 3a,b. No imaginary frequencies are observed throughout the whole Brillouin zone, confirming the dynamical stability of *Pnma*-BN.

In an effort to assess the thermodynamic stability of *Pnma*-BN, enthalpy change curves with pressure for various structures were calculated, as presented in Figure 3c. The dashed line represents the enthalpy of the $F\bar{4}3m$-BN (c-BN). It can be clearly seen that *P*6_3_/*mmc*-BN has the lowest minimum value of enthalpy, which is in good agreement with previous reports and supports the reliability of our calculations. The minimum value of total energy per formula unit of BN is slightly larger than that of *Pbam*-BN and *P*6_3_/*mc*-BN, hence *Pnma*-BN should be thermodynamically metastable.

### 3.3. Mechanical and Anisotropic Properties

**Figure 4:**
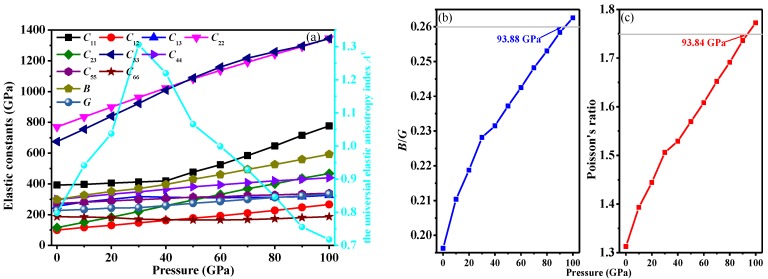
Elastic constants and elastic modulus (**a**) and *B*/*G* ratio (**b**); Poissons’ ratio *v* (**c**) of *Pnma*-BN as a function of pressure.

The elastic constants and elastic modulus of *Pnma*-BN as a function of pressure are shown in Figure 4a, all elastic constants and elastic modulus of *Pnma*-BN are increasing with different rates as pressure increases, except for *C*_66_. It is well known that bulk modulus (*B*) represents the resistance to material fracture, whereas the shear modulus (*G*) represents the resistance to plastic deformation of a material, Young’s modulus (*E*) describes tensile elasticity. Young’s modulus *E* and Poisson’s ratio *v* are taken as: *E* = 9*BG*/(3*B* + *G*), *v* = (3*B* − 2*G*)/[2(3*B* + *G*)].

Hence, the Pugh ratio (*B*/*G* ratio) is defined as a quantitative index for assessing the brittle or ductile behavior of crystals. According to Pugh [43], a larger *B*/*G* value (*B*/*G* > 1.75) for a solid represents ductile, while a smaller *B*/*G* value (*B*/*G* < 1.75) usually means brittle. Moreover, Poisson’s ratio *v* is consistent with *B*/*G*, which refers to ductile compounds usually with a large *v* (*v* > 0.26) [44]. The value of Poisson’s ratio *v* and *B*/*G* various pressure as functions for *Pnma*-BN are shown in Figure 4b,c, respectively, which indicates that *Pnma*-BN is brittle when pressure less than around 94 GPa. The values of *B*/*G* and *v* for *Pnma*-BN are 1.312 and 0.196 at ambient pressure, respectively. *Pnma*-BN is found to turn from brittle to ductile in this pressure range (0–100 GPa).

Based on elastic modulus and other related values, the hardness (*H_v_*) of *Pnma*-BN are evaluated using two different empirical models: Chen et al. model [45] and Lyakhov and Oganov’s et al. model [46,47], the calculated results of Chen et al. model and Ma et al. model are 31.8 GPa and 33.3 GPa. The results of Chen et al. model are slightly smaller than that of Lyakhov and Oganov’s model. The main reason for this situation is that an empirical formula may estimate the value of the material’s hardness as too high or too low. Most researchers agree on the definition according to which “superhard” materials are those with *H*_v_ exceeding 40 GPa [15]. Although there are slightly differences between the results of the two empirical models above, the hardness of *Pnma*-BN is slightly smaller than 40 GPa, indicating that *Pnma*-BN is a hard material.

The Poisson’s ratio *v*, shear modulus *G* and Young’s modulus *E* may have different values depending on the direction of the applied force with respect to the structure, so we continued to investigate the mechanical anisotropy properties of *Pnma*-BN. A fourth order tensor transforms in a new basis set following the rule:

(6) $$S_{\alpha \beta \gamma \delta }^{\prime }=r_{ai}r_{aj}r_{ak}r_{al}S_{ijkl}$$

where Einstein’s summation rule is adopted and where the *r*_αi_ is the component of the rotation matrix (or direction cosines). The Young’s modulus can be obtained by using a purely normal stress in ε*_ij_* = *S*_ijkl_σ_kl_ in its vector form and it is given by the following form:

(7) $$E(\theta ,\varphi )=\frac{1}{S_{11}^{\prime }(\theta ,\varphi )}=\frac{1}{r_{1i}r_{1j}r_{1k}r_{1l}S_{ijkl}}=\frac{1}{a_{i}a_{j}a_{k}a_{l}S_{ijkl}}$$

The Poisson’s ratio and shear modulus depending on two directions (if perpendicular, this corresponds to three angles) make them difficult to represent graphically. A convenient possibility is then to consider three representations: minimum, average, and maximum. For each θ and ϕ, the angle χ is scanned and the minimum, average, and maximum values are recorded for this direction. The transformation can be substantially simplified in calculation of specific modulus. The uniaxial stress can be represented as a unit vector, and advantageously described by two angles θ, ϕ, we choose it to be the first unit vector in the new basis set ***a***. The determination of some elastic properties (shear modulus, Poisson’s ratio) requires another unit vector ***b***, perpendicular to unit vector ***a***, and characterized by the angle χ. It is fully characterized by the angles θ (0, π), ϕ (0, 2π), and χ (0, 2π), as illustrated in Reference [48]. The coordinates of two vectors are:

(8) $$a=\left(\begin{array}{c} \sin\theta \cos\varphi \\ \sin\theta \sin\varphi \\ \cos\theta \end{array}\right)\;b=\left(\begin{array}{c} \cos\theta \cos\varphi \cos\chi -\sin\varphi \sin\chi \\ \cos\theta \sin\varphi \cos\chi +\cos\varphi \sin\chi \\ -\sin\theta \cos\chi \end{array}\right)$$

The shear modulus in the vector form is obtained by applying a pure shear stress, then it can be expressed as:

(9) $$G(\theta ,\varphi ,\chi )=\frac{1}{4S_{66}^{\prime }(\theta ,\varphi ,\chi )}=\frac{1}{4r_{1i}r_{2j}r_{1k}r_{2l}S_{ijkl}}=\frac{1}{4a_{i}a_{j}a_{k}a_{l}S_{ijkl}}$$

The Poisson’s ratio can be given in:

(10) $$v(\theta ,\varphi ,\chi )=-\frac{S_{12}^{\prime }(\theta ,\varphi ,\chi )}{S_{11}^{\prime }(\theta ,\varphi )}=-\frac{r_{1i}r_{1j}r_{2k}r_{2l}S_{ijkl}}{r_{1i}r_{1j}r_{1k}r_{1l}S_{ijkl}}=-\frac{a_{i}a_{j}b_{k}b_{l}S_{ijkl}}{a_{i}a_{j}a_{k}a_{l}S_{ijkl}}$$

**Figure 5:**
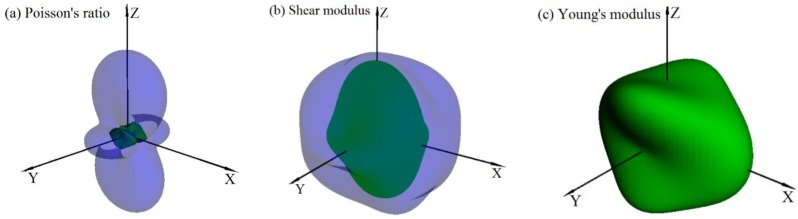
The surface construction of Poisson’s ratio (**a**); shear modulus (**b**); and Young’s modulus (**c**) for the *Pnma*-BN.

The three-dimension surface representation of Poisson’s ratio *v*, shear modulus *G*, and Young’s modulus *E* for *Pnma*-BN are illustrated in Figure 5a–c, respectively. The green and purple surface representation denoted the minimum and the maximum values of Poisson’s ratio *v* and shear modulus *G*, respectively. For an isotropic system, the three-dimension directional dependence would exhibit a spherical shape, while the deviation degree from the spherical shape reflects the content of anisotropy [49]. From Figure 5a–c, one can note that the Poisson’s ratio, shear modulus, and Young’s modulus show different degree anisotropy of *Pnma*-BN. *Pnma*-BN shows the largest anisotropy in Poisson’s ratio than that of shear modulus and Young’s modulus.

**Figure 6:**
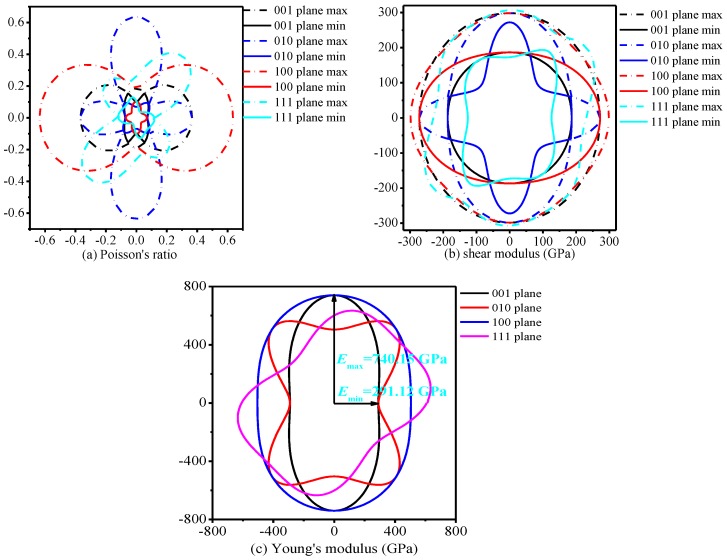
2D representation of Poisson’s ratio (**a**); shear modulus (**b**) and Young’s modulus (**c**) in the main plane for *Pnma*-BN, respectively.

In order to investigate the anisotropy of *Pnma*-BN in detail, the two-dimension representations of the shear modulus in the (001) plane, (010) plane, (100) plane, and (111) plane for *Pnma*-BN are illustrated in Figure 6a–c, and the calculated the maximum and minimum values of Poisson’s ratio *v*, shear modulus *G* and Young’s modulus *E* for *Pnma*-BN are listed in Table 3. From Figure 6a–c and Table 3, one can find that *Pnma*-BN shows a larger anisotropy in Poisson’s ratio *v*, shear modulus *G* and Young’s modulus *E*. For Poisson’s ratio *v*, the maximum value appears in the (010) and (100) planes, the minimum value are not appearing in these planes, but (100) plane (*v*_max_/*v*_min_ = 14.431) shows the largest anisotropy. (100) plane shows the largest anisotropy in Poisson’s ratio *v*, while it shows the smallest anisotropy in shear modulus and Young’s modulus. For shear modulus, the maximum value of all directions for shear modulus is 310.85 GPa, while maximum values are not appearing in the (001), (100), (010), and (111) planes, the minimum value appear in the (010) and (111) planes. The anisotropies of shear modulus in all directions for *Pnma*-BN reduce in the sequence of (111) plane > (010) plane > (001) plane = (100) plane. The maximum value of Young’s modulus appears in the (001) and (100) planes, while the minimum value appears in the (010) and (100) planes, so the (001) plane shows the largest anisotropy in Young’s modulus. The anisotropies of Young’s modulus in all directions for *Pnma*-BN follow the order: (001) plane > (010) plane > (111) plane > (100) plane.

The universal elastic anisotropy index *A*^U^ proposes an anisotropy measure based on the Reuss and Voigt averages which quantifies the single crystal elastic anisotropy, and *A*^U^ = 5*G_V_*/*G_R_* + *B_V_*/*B_R_* − 6 [50]. The universal elastic anisotropy index *A*^U^ as a function of pressure is shown in Figure 4a. The universal elastic anisotropy index *A*^U^ increases with increasing pressure from 0 to 30 GPa, then it decreases with increasing pressure with 30 to 100 GPa. At ambient pressure, *Pnma*-BN has a larger universal elastic anisotropy index *A*^U^ (0.798). It is almost eight times that of *Pbca*-BN (0.095).

The interest in the calculation of the Debye temperature Θ_D_ has been increasing in both semiempirical and theoretical phase diagram calculation areas since the Debye model offers a simple but highly efficiency method to describe the phonon contribution to the Gibbs energy of crystalline phases. The average sound velocity *v_m_* and Debye temperature Θ_D_ can be approximately calculated by the following relations [51]:

(11) $$\Theta_{D}=\frac{h}{k_{B}}{\left[\frac{3n}{4\pi }(\frac{N_{A}\rho }{M})\right]}^{\frac{1}{3}}v_{m}$$

(12) $$v_{m}=\frac{1}{3}\sum_{i=1}^{3}\int \frac{1}{v_{i}^{3}(\theta ,\varphi )}\frac{d\Omega }{4\pi }={\left[\frac{1}{3}(\frac{2}{v_{l}^{3}}+\frac{1}{v_{t}^{3}})\right]}^{-\frac{1}{3}}$$

*v*_l_ and *v_t_* are the longitudinal and transverse sound velocities, respectively, which can be obtained from Navier’s equation [52]:

(13) $$v_{l}=\sqrt{(B+\frac{4}{3}G)\frac{1}{\rho }}\;v_{t}=\sqrt{\frac{G}{\rho }}$$

where *h* is Planck’s constant, *k_B_* is Boltzmann’s constant, *N*_A_ is Avogadro’s number, *n* is the number of atoms in the molecule, *M* is molecular weight, and ρ is the density, (θ, ϕ) are angular coordinates and dΩ = sinθdθdϕ. If the elastic constants of the crystal are known, *v_i_* (θ, ϕ) can be obtained by solving a secular equation, and *v_m_* and Θ_D_ can then be calculated by numerical integration over θ and ϕ [53,54]. The calculated sound velocities and Debye temperatures under pressure of *Pnma*-BN are listed in Table 4. The Debye temperature of *Pnma*-BN is 1502 K, it is smaller than that of *Pbca*-BN (Θ_D_ = 1734 K) at ambient pressure, and it is also smaller than $F\bar{4}3m$-BN (Θ_D_ = 1896 K), the result of $F\bar{4}3m$-BN has a high credibility [55]. The longitudinal and transverse sound velocities of *Pnma*-BN are smaller than *Pbca*-BN [39] and $F\bar{4}3m$-BN, because *Pnma*-BN has the smaller elastic modulus.

### 3.4. Electronic Properties

**Figure 7:**
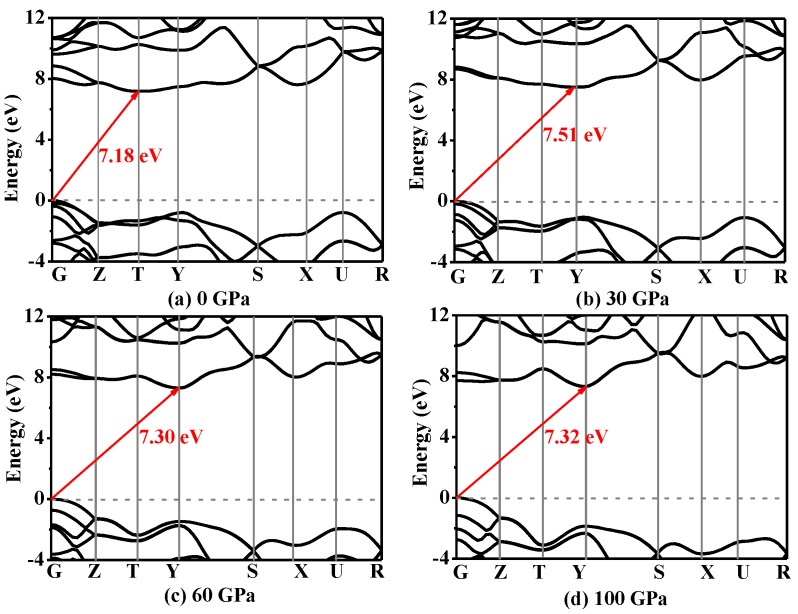
The band structures under pressure of *Pnma*-BN, (**a**) 0 GPa, (**b**) 30 GPa, (**c**) 60 GPa, (**d**) 100 GPa.

**Figure 8:**
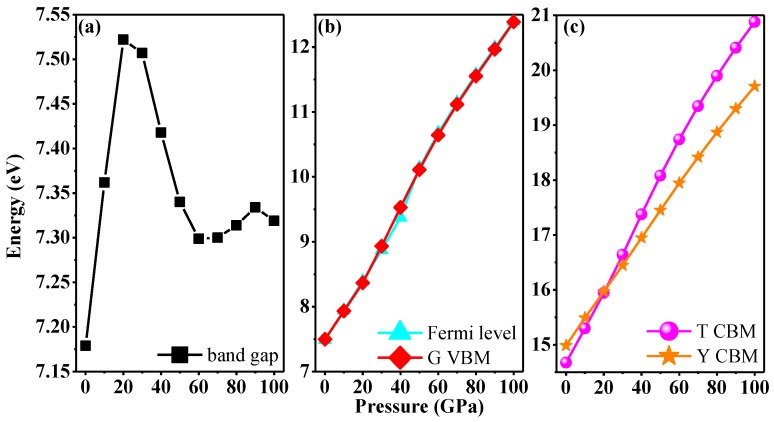
The band gap under pressure of *Pnma*-BN (**a**), the Fermi level and the energy of *G* high-symmetry points along valence band maximum (VBM) (**b**); the energy of *T* and *Y* high-symmetry points along conduction band minimum (CBM) (**c**).

The band structures with Heyd–Scuseria–Ernzerhof (HSE06) hybrid-functional [56,57] along high-symmetry direction in Brillouin zone under pressure of *Pnma*-BN are shown in Figure 7. At ambient pressure, *Pnma*-BN is an insulator with band gap of 7.18 eV. The band gap of *Pnma*-BN is slightly larger than that of h-BN at ambient pressure (LDA: 4.01 eV [58], Experiment: 5.97 eV [59]). When *p* = 30 GPa, the band gap of *Pnma*-BN is 7.51 eV, while the band gap is 7.30 eV when *p* = 60 GPa. More interestingly, with pressure increasing to 100 GPa, the band gap increases to 7.32 eV. Unusually, the band gap of *Pnma*-BN is not monotonically increasing or monotonically decreasing with increasing pressure. The band gap of *Pnma*-BN as a function of pressure is shown in Figure 8a. From 0 to 60 GPa, the band gap increases first and then decreases with the increase of pressure, and from 60 to 100 GPa, the band gap increases first and then decreases.

Figure 8b,c shows the energies of Fermi level and *G* high-symmetry point along valence band maximum (VBM), the energies of *T* and *Y* high-symmetry points along conduction band minimum (CBM) as functions with pressure, respectively. From Figure 8b, it is clear that the Fermi levels are very close to *G* high-symmetry point along VBM. The energies of *T* and *Y* high-symmetry points along CBM both increase with increasing pressure. From 0 to 20 GPa, the energy of *Y* high-symmetry points along CBM is greater than that of *T* high-symmetry points, while when *p* = 20 GPa, the energy of *Y* high-symmetry points along CBM (15.97 eV) is very close to *T* high-symmetry points (15.94 eV). With increasing pressure (from 20 to 100 GPa), the energy of *T* high-symmetry points along CBM is greater than that of *Y* high-symmetry points (see Figure 7b–d).

## 4. Conclusions

The calculated lattice parameters agree very well with reported values in the literature, for all phases of both materials. The *Pnma* phase of BN is found to be metastable. The calculated Pugh ratio and Poisson’s ratio revealed that *Pnma*-BN is brittle, and *Pnma*-BN is found to turn from brittle to ductile (~94 GPa) in this pressure range. In addition, the mechanical anisotropy properties of *Pnma*-BN are investigated in this paper. *Pnma*-BN shows a larger anisotropy in Poisson’s ratio *v*, shear modulus *G* and Young’s modulus *E*, and its anisotropy is greater than that of *Pbca*-BN and $F\bar{4}3m$-BN. The calculated band structure revealed that *Pnma*-BN is an insulator with band gap of 7.18 eV at ambient pressure. More interesting, the band gap of *Pnma*-BN is not monotonically increasing or monotonically decreasing with increasing pressure. From 0 to 60 GPa, the band gap increases first and then decreases with the increase of pressure, and from 60 to 100 GPa, the band gap increases first and then decreases. In addition, we will study nitride boron nitride (BN), aluminum nitride (AlN), and gallium nitride (GaN) [60] alloys, mainly researching some physical properties, such as mechanical properties, electronic properties, and mechanical anisotropy properties.

## Figures and Tables

**Table 1:** The calculated lattice parameters of BN polymorphs.

Space Group	Methods	*a* (Å)	*b* (Å)	*c* (Å)	*V* (Å^3^)
*Pnma*	GGA	4.8900	2.5890	4.2835	13.5574
	LDA	4.7954	2.5569	4.2432	13.0068
	LDA ^1^	4.7600	2.5800	4.2900	13.1712
*Pbca*	GGA	5.0987	4.4216	4.3981	12.3940
	GGA ^2^	5.1103	4.4336	4.3992	12.4591
	LDA	5.0412	4.3794	4.3316	11.9538
	LDA ^2^	5.0458	4.3800	4.3392	11.9873
$F\bar{4}3m$	GGA	3.6258			11.9166
	GGA ^3^	3.6224			11.8835
	LDA	3.5692			11.3672
	LDA ^3^	3.5764			11.4364
	Experiment ^4^	3.6200			11.8595

^1^ Reference [38]; ^2^ Reference [39]; ^3^ Reference [40]; ^4^ Reference [41].

**Table 2:** The calculated elastic constants (GPa) and elastic modulus (GPa) within GGA level of *Pnma*-BN, *Pbca*-BN, and h-BN.

Materials	*p*	*C*_11_	*C*_12_	*C*_13_	*C*_22_	*C*_23_	*C*_33_	*C*_44_	*C*_55_	*C*_66_	*B*	*G*	*E*	*v*
*Pnma*-BN	0	392	99	256	770	116	675	299	272	187	298	227	543	0.196
	0 ^1^	403	107	273	824	132	730	316	282	187	318	236	568	0.202
	10	397	117	282	836	150	756	316	281	185	326	234	566	0.210
	20	405	131	300	899	184	840	333	289	181	351	243	592	0.219
	30	412	147	318	961	220	923	348	297	171	369	245	602	0.228
	40	420	162	316	1023	258	1010	365	305	167	396	259	638	0.232
	50	477	177	311	1083	295	1089	381	314	165	430	274	678	0.237
	60	524	192	310	1138	331	1159	394	315	166	460	286	711	0.242
	70	584	209	312	1191	368	1217	408	325	168	494	299	746	0.248
	80	646	227	314	1243	400	1259	420	329	173	526	311	779	0.253
	90	716	247	317	1292	437	1296	430	334	181	559	322	810	0.258
	100	777	266	326	1347	468	1344	440	339	187	592	334	843	0.263
*Pbca*-BN	0	769	145	133	870	105	716	307	255	340	340	312	717	0.148
	0 ^2^	772	135	139	885	92	716	312	257	357	344	316	718	0.140
c-BN	0	788	160				443				369	386	859	0.112
	0 ^2^	779	165				446				370	384	856	0.120

^1^ local density approximation (LDA) level; ^2^ Reference [39].

**Table 3:** The calculated the maximum and minimum values of Poisson’s ratio *v*, Shear modulus *G*, and Young’s modulus *E* for *Pnma*-BN.

Surface	Poisson’s Ratio *v*			Shear Modulus (GPa)			Young’s Modulus (GPa)		
	*v*_max_	*v*_min_	*v*_max_/*v*_min_	*G*_max_	*G*_min_	*G*_max_/*G*_min_	*E*_max_	*E*_min_	*E*_max_/*E*_min_
(001)	0.366	0.074	4.945	298.60	186.70	1.599	740.15	291.12	2.534
(010)	0.635	0.069	9.203	298.60	126.04	2.369	658.13	291.12	2.261
(100)	0.635	0.044	14.431	298.60	186.70	1.599	740.15	504.52	1.467
(111)	0.494	0.045	10.978	307.33	126.04	2.439	649.49	389.52	1.667
All	0.635	0.010	63.500	310.85	126.04	2.466	740.15	291.12	2.534

**Table 4:** The calculated density ρ (g/cm^3^), sound velocities (m/s), and Debye temperature (*K*) of *Pnma*-BN.

*p*	ρ	*v_p_*	*v_s_*	*v_m_*	Θ_D_
0	3.040	14057	8642	9537	1502
10	3.144	14244	8627	9534	1518
20	3.248	14415	8649	9567	1540
30	3.354	14402	8547	9465	1540
40	3.460	14639	8653	9585	1576
50	3.559	14949	8774	9727	1614
60	3.653	15176	8848	9815	1643
70	3.740	15449	8941	9924	1674
80	3.822	15688	9020	10018	1702
90	3.899	15920	9087	10098	1728
100	3.973	16158	9169	10194	1755

## References

[1] Tian Y.J., Xu B., Yu D.L., Ma Y.M., Wang Y.B., Jiang Y.B., Hu W.T., Tang C.C., Gao Y.F., Luo K. (2013). Ultrahard nanotwinned cubic boron nitride. Nature.

[2] Zhang M.G., Wei Q., Yan H.Y., Zhao Y.R., Wang H. (2014). A novel superhard tetragonal carbon mononitride. J. Phys. Chem. C.

[3] Wang X.L. (2012). Polymorphic phases of sp^3^-hybridized superhard CN. J. Chem. Phys..

[4] Fan Q.Y., Wei Q., Yan H.Y., Zhang M.G., Zhang D.Y., Zhang J.Q. (2014). A new potential superhard phase of OsN_2_. Acta Phys. Pol. A.

[5] Yan H.Y., Zhang M.G., Wei Q., Guo P. (2013). *Ab initio* studies of ternary semiconductor BeB_2_C_2_. Comput. Mater. Sci..

[6] Pease R.S. (1952). An X-ray study of boron nitride. Acta Crystallogr..

[7] Bundy P., Wentorf R.H. (1963). Direct transformation of hexagonal boron nitride to denser forms. J. Chem. Phys..

[8] Thomas J., Weston N.E., Oconnor T.E. (1963). Turbostratic boron nitride, thermal transformation to ordered-layer-lattice boron nitride. J. Am. Chem. Soc..

[9] Mirkarimi P.B., McCarty K.F., Medlin D.L. (1997). Review of advances in cubic boron nitride film synthesis. Mater. Sci. Eng. R-Rep..

[10] Paine R.T., Narula C.X. (1990). Synthetic routes to boron nitride. Chem. Rev..

[11] Bosak A., Serrano J., Krisch M., Watanabe K., Taniquchi T., Kanda H. (2006). Lateral adsorption geometry and site-specific electronic structure of a large organic chemisorbate on a metal surface. Phys. Rev. B.

[12] Novoselov K.S., Jiang D., Schedin F., Booth T.J., Khotkevich V.V., Morozov S.V., Geim A.K. (2005). Two-dimensional atomic crystals. Proc. Natl. Acad. Sci. USA.

[13] Chopra N.G., Luyken R.J., Cherrey K., Crespi V.H., Cohen M.L., Louie S.G., Zettl A. (1995). Boron Nitride Nanotubes. Science.

[14] Jiang X., Zhao J.J., Ahuja R. (2013). A novel superhard BN polymorph under cold compression of h-BN. J. Phys. Condens. Matter..

[15] Zhang X.X., Wang Y.C., Lv J., Zhu C.Y., Li Q., Zhang M., Li Q., Ma Y.M. (2013). First-principles structural design of superhard materials. J. Chem. Phys..

[16] Zhang S.H., Wang Q., Kawazoe Y., Jena P.R. (2013). Three-dimensional metallic boron nitride. J. Am. Chem. Soc..

[17] He C.Y., Sun L.Z., Zhang C.X., Peng X.Y., Zhang K.W., Zhong J.X. (2012). Z-BN: A novel superhard boron nitride phase. Phys. Chem. Chem. Phys..

[18] Yang G., Chen B.F. (2014). Predicted a novel high-pressure superhard boron nitride phase. J. Alloy Compd..

[19] Niu C.Y., Wang J.T. (2014). Three-dimensional three-connected tetragonal BN: *Ab initio* calculations. Phys. Lett. A.

[20] Germaneau E., Su G., Zheng Q.R. (2013). New boron nitride structures B_4_N_4_: A first-principles random searching application. J. Phys. Condens. Matter..

[21] Zhang Z.G., Lu M.C., Zhu L., Zhu L.L., Li Y.D., Zhang M., Li Q. (2014). Orthorhombic BN: A novel superhard image boron nitride polymorph. Phys. Lett. A.

[22] Wen B., Zhao J.J., Melnikc R., Tian Y.J. (2011). Body-centered tetragonal B_2_N_2_: A novel sp^3^ bonding boron nitride polymorph. Phys. Chem. Chem. Phys..

[23] Dai J., Wu X., Yang J., Zeng X.C. (2013). Unusual metallic microporous boron nitride networks. J. Phys. Chem. Lett..

[24] Dai J., Wu X., Yang J., Zeng X.C. (2014). Porous boron nitride with tunable pore size. J. Phys. Chem. Lett..

[25] Lian J.B., Kim T., Liu X.D., Ma J.M., Zheng W.J. (2009). Ionothermal Synthesis of Turbostratic Boron Nitride Nanoflakes at Low Temperature. J. Phys. Chem. C.

[26] Li Y.W., Hao J., Liu H.Y., Lu S.Y., Tse J.S. (2015). High-energy density and superhard nitrogen-rich B-N compounds. Phys. Rev. Lett..

[27] Perdew J.P., Burke K., Ernzerhof M. (1996). Generalized gradient approximation made simple. Phys. Rev. Lett..

[28] Perdew J.P., Zunger A. (1981). Self-interaction correction to density-functional approximations for many-electron systems. Phys. Rev. B.

[29] Clark S.J., Segall M.D., Pickard C.J., Hasnip P.J., Probert M.I.J., Refson K., Payne M.C. (2005). First principles methods using CASTEP. Z. Kristallogr..

[30] Pfrommer B.G., Côté M., Louie S.G., Cohen M.L. (1997). Relaxation of crystals with the Quasi-Newton method. J. Comput. Phys..

[31] Vanderbilt D. (1990). Soft self-consistent pseudopotentials in a generalized eigenvalue formalism. Phys. Rev. B.

[32] Monkhorst H.J., Pack J.D. (1976). Special points for Brillouin-zone integrations. Phys. Rev. B.

[33] Fan Q.Y., Chai C.C., Wei Q., Yang Y.T., Yang Q., Chen P.Y., Xing M.J., Zhang J.Q., Yao R.H. (2016). Prediction of novel phase of silicon and Si–Ge alloys. J. Solid State Chem..

[34] Fan Q.Y., Chai C.C., Wei Q., Yang Y.T., Qiao L.P., Zhao Y.B., Zhou P.K., Xing M.J., Zhang J.Q., Yao R.H. (2015). Mechanical and electronic properties of Ca_1-*x*_Mg*_x_*O alloys. Mater. Sci. Semicond. Process.

[35] Voigt W. (1928). Lehrburch der Kristallphysik.

[36] Reuss A. (1929). Berechnung der Fließgrenze von Mischkristallen auf Grund der Plastizitätsbedingung für Einkristalle. J. Appl. Math. Mech..

[37] Hill R. (1952). The elastic behaviour of a crystalline aggregate. Phys. Soc. Lond. Sect. A.

[38] Doll K., Schön J.C., Jansen M. (2008). Structure prediction based on *ab initio* simulated annealing for boron nitride. Phys. Rev. B.

[39] Fan Q.Y., Wei Q., Yan H.Y., Zhang M.G., Zhang Z.X., Zhang J.Q., Zhang D.Y. (2014). Elastic and electronic properties of Pbca-BN: First-principles calculations. Comput. Mater. Sci..

[40] Fan Q.Y., Wei Q., Chai C.C., Yan H.Y., Zhang M.G., Lin Z.Z., Zhang Z.X., Zhang J.Q., Zhang D.Y. (2015). Structural, mechanical, and electronic properties of P3m1-BCN. J. Phys. Chem. Solids.

[41] Petrescu M.L. (2004). Boron nitride theoretical hardness compared to carbon polymorphs. Diamond Relat. Mater..

[42] Wu Z.J., Zhao E.J., Xiang H.P., Hao X.F., Liu X.J., Meng J. (2007). Crystal structures and elastic properties of superhard IrN_2_ and IrN_3_ from first principles. Phys. Rev. B.

[43] Pugh S.F. (1954). XCII. Relations between the elastic moduli and the plastic properties of polycrystalline pure metals. Lond. Edinb. Dublin Philos. Mag. J. Sci..

[44] Duan Y.H., Sun Y., Peng M.J., Zhou S.G. (2014). Anisotropic elastic properties of the Ca–Pb compounds. J. Alloy. Compd..

[45] Chen X.-Q., Niu H., Li D., Li Y. (2011). Modeling hardness of polycrystalline materials and bulk metallic glasses. Intermetallics.

[46] Lyakhov A.O., Oganov A.R. (2011). Evolutionary search for superhard materials: Methodology and applications to forms of carbon and TiO_2_. Phys. Rev. B.

[47] Xing M.J., Li B.H., Yu Z.T., Chen Q. (2016). Monoclinic *C*2/*m*-20 carbon: A novel superhard *sp*^3^ carbon allotrope. RSC Adv..

[48] Marmier A., Lethbridge Z.A.D., Walton R.I., Smith C.W., Parker S.C., Evans K.E. (2010). ElAM: A computer program for the analysis and representation of anisotropic elastic properties. Comput. Phys. Commun..

[49] Hu W.C., Liu Y., Li D.J., Zeng X.Q., Xu C.S. (2014). First-principles study of structural and electronic properties of C14-type Laves phase Al_2_Zr and Al_2_Hf. Comput. Mater. Sci..

[50] Ranganathan S.I., Ostoja-Starzewski M. (2008). Universal elastic anisotropy index. Phys. Rev. Lett..

[51] Anderson O.L. (1963). A simplified method for calculating the debye temperature from elastic constants. J. Phys. Chem. Solids..

[52] Panda K.B., Ravi K.S. (2006). Determination of elastic constants of titanium diboride (TiB_2_) from first principles using FLAPW implementation of the density functional theory. Comput. Mater. Sci..

[53] Anderson O.L. (1965). Physical Acoustics.

[54] Grimvall G. (1986). Thermophysical Properties of Materials.

[55] Adachi S. (2004). Handbook on Physical Properties of Semiconductors.

[56] Heyd J., Scuseria G.E., Ernzerhof M. (2003). Hybrid functionals based on a screened Coulomb potential. J. Chem. Phys..

[57] Fan Q., Chai C., Wei Q., Yang Y. (2016). Two novel silicon phases with direct band gaps. Phys. Chem. Chem. Phys..

[58] Ohba N., Miwa K., Nagasako N., Fukumoto A. (2001). First-principles study on structural, dielectric, and dynamical properties for three BN polytypes. Phys. Rev. B.

[59] Watanabe K., Taniguchi T., Kanda H. (2004). Direct-bandgap properties and evidence for ultraviolet lasing of hexagonal boron nitride single crystal. Nat. Mater..

[60] Fan Q., Chai C., Wei Q., Yang J., Zhou P., Zhang D., Yang Y. (2016). A new phase of GaN. J. Chem..

